# Inactivation of Pathogens in Air Using Ultraviolet Direct Irradiation Below Exposure Limits

**DOI:** 10.6028/jres.126.052

**Published:** 2022-03-01

**Authors:** Gary R. Allen, Kevin J. Benner, William P. Bahnfleth

**Affiliations:** 1 Gary Allen Consulting, Inc., Euclid, OH 44119, USA; 2 GE Current, a Daintree company, East Cleveland, OH 44112, USA; 3 Department of Architectural Engineering The Pennsylvania State University State College, PA 16801, USA

**Keywords:** actinic, airborne, disinfection, exposure limits, germicidal, indoor air quality, LED, light-emitting diodes, pathogen, SARS-CoV-2, ultraviolet, UV-C, virus

## Abstract

A method is described for inactivation of pathogens, especially airborne pathogens, using ultraviolet (UV) radiation emitted directly into occupied spaces and exposing occupants to a dose below the accepted actinic exposure limit (EL). This method is referred to as direct irradiation below exposure limits, or DIBEL. It is demonstrated herein that low-intensity UV radiation below exposure limits can achieve high levels of equivalent air changes per hour (ACH_eq_) and can be an effective component of efforts to combat airborne pathogens such as the severe acute respiratory syndrome coronavirus 2 (SARS-CoV-2), the virus that causes coronavirus disease 2019 (COVID-19). An ACH_eq_ of 4 hˉ¹ is presently achievable over a continuous 8 h period for the SARS-CoV-2 virus with UV-C light-emitting diodes (LEDs) having peak wavelength at 275 nm, and future improvements in LED technology and optics are anticipated to enable improvements up to 150 hˉ¹ in the coming decade. For example, the actinic EL is 60 J/m² at 254 nm, and human coronaviruses, including SARS-CoV-2, have a UV dose required for 90 % inactivation of about 5 J/m² at 254 nm. Irradiation by 254 nm UV-C at the EL is expected to provide 90 % inactivation of these organisms in air in about 40 min when the UV-C is delivered at a constant irradiance over 8 h, or in about 5 min if the UV-C is delivered at a constant irradiance over 1 h. Since the irradiation is continuous, the inactivation of initial contaminants accumulates to 99 % and then 99.9 %, and it also immediately begins inactivating any newly introduced (e.g., exhaled) pathogens at the same rate throughout the 8 h period. The efficacy for inactivating airborne pathogens with DIBEL may be expressed in terms of ACHeq, which may be compared with conventional ventilation-based methods for air disinfection. DIBEL may be applied in addition to other disinfection methods, such as upper room UV germicidal irradiation, and mechanical ventilation and filtration. The ACHeq of the separate methods is additive, providing enhanced cumulative disinfection rates. Conventional air disinfection technologies have typical ACH_eq_ values of about 1 hˉ¹ to 5 hˉ¹ and maximum practical values of about 20 hˉ¹. UV-C DIBEL currently provides ACH_eq_ values that are typically about 1 hˉ¹ to 10 hˉ¹, thus either complementing, or potentially substituting for, conventional technologies. UV-C DIBEL protocols are forecast herein to evolve to >100 ACH_eq_ in a few years, potentially surpassing conventional technologies. UV-A (315 nm to 400 nm) and/or UV-C (100 nm to 280 nm) DIBEL is also efficacious at inactivating pathogens on surfaces. The relatively simple installation, low acquisition and operating costs, and unobtrusive aesthetic of DIBEL using UV LEDs contribute value in a layered, multi-agent disinfection strategy.

This article was sponsored by Dianne L. Poster, Material Measurement Laboratory, and C. Cameron Miller, Physical Measurement Laboratory, National Institute of Standards and Technology (NIST). It is published in collaboration with the International Ultraviolet Association as a complement to the NIST Workshop on Ultraviolet Disinfection Technologies, 14–15 January 2020, Gaithersburg, MD. The views expressed represent those of the authors and not necessarily those of NIST.

## 1 Introduction

Ultraviolet (UV) radiation has been known to inactivate microorganisms since at least the late 1800s. It was demonstrated to disinfect water in 1878 [1]. Niels Finsen received the Nobel Prize in Medicine in 1903 for the use of UV in skin disease treatment, in particular, lupus vulgaris, which is caused primarily by *Mycobacterium tuberculosis* [2]. Over the last several decades, hundreds of photobiological studies have determined the sensitivities of a wide array of bacterial, fungal, and viral organisms to UV, particularly UV-C [3].

Repeated and acute UV exposure at certain doses poses a risk to humans, particularly to skin and eye tissue. It is thus important to define exposure limits (ELs)—the daily doses below which there is no expectation of photobiological harm from repeated exposure. Two examples of such definitions are the Ultraviolet Radiation Threshold Limit Values (TLVs^®^)^1^1 Certain commercial instruments and materials are identified to specify the experimental study adequately. This does not implyrecommendation or endorsement by the National Institute of Standards and Technology, nor does it imply that the instruments andmaterials identified are necessarily the best available for the purpose. published by the American Conference of Governmental Industrial Hygienists (ACGIH) [4] and the ELs given in the Commission Internationale de l’Eclairage’s international standard *Photobiological Safety of Lamps and Lamp Systems* [5].

Although the dose required to achieve a 1 log_10_ (90%) or higher level of inactivation of many pathogens is less than the ELs [3], many typical UV disinfection applications use high-intensity UV sources such as discharge lamps that can achieve high levels of disinfection in seconds or minutes, but they also exceed the ELs by orders of magnitude in very short periods of time.^2^2 1 log_10_ units refers to a 90% reduction, calculated as log_10_ (*N*_0_/*N*), where *N*_0_ is the initial value, and *N* is the final value. This makes them unsuitable for direct exposure of the occupied zone of spaces, although upper room systems that create a high-intensity disinfection zone above the occupied zone have been in use since the 1930s [6].

The recent advent of low-intensity UV light sources such as UV light-emitting diodes (LEDs) [7] and low-power excimer lamps [8] has enabled the irradiation of UV directly into an occupied space at irradiances below the allowed limits for human exposure to UV, herein referred to as direct irradiation below exposure limits (DIBEL). DIBEL differs from upper room systems by directly irradiating and disinfecting the space while occupied, without the need for forced or natural convection to move air from the upper room to the occupied zone; therefore, it is not limited by the room air flow patterns nor ventilation rate of the air. Previously, to directly irradiate the occupied zone below the EL with conventional high-intensity UV sources, such a source would need to be mounted several meters away from the occupied zone (not possible in most indoor spaces) or heavily reduced in time-averaged irradiance either by reducing power, filtering, or pulsing.

**Fig. 1:**
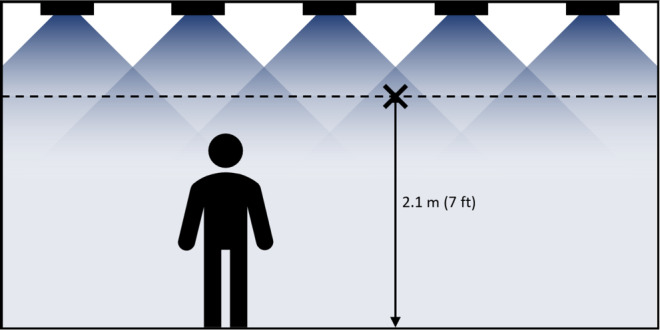
Typical application of DIBEL technology in an occupied room.

DIBEL technology can achieve significant levels of pathogen inactivation (*e.g.*, >50% in an hour or less) by providing direct, continuous radiation into the occupied breathing zone while adhering to actinic dose ELs. While DIBEL with UV-C is shown herein to be efficacious for airborne pathogens while the space is occupied, DIBEL can be supplemented by additional irradiation exceeding the ELs into unoccupied spaces, and by additional irradiation onto surfaces to reduce fomite contamination. An exemplary DIBEL installation may comprise an array of UV LEDs emitting a narrow spectrum with a peak wavelength that is efficacious at inactivating one or more target pathogens in the air and/or on surfaces. The array may be mounted on or near the ceiling of an occupied space, such as an office, conference room, restaurant, *etc*., providing nearly uniform irradiance throughout the occupied space, not to exceed the maximum allowed EL at 2.1 m (7 ft) above the floor over an 8 h period [5]. A typical layout is shown schematically in Fig. 1, where a two-dimensional array of equally spaced emitters (represented by black rectangles) is mounted on the ceiling radiating downward. UV radiation is represented in blue, where darker color represents higher irradiance. The UV irradiance resulting from any one emitter is the greatest when measured close to the device and decreases as distance from the device increases. At locations that are not very close to a single device, the irradiance at a given location results from contributions from many devices. Because radiated energy is only lost when it is absorbed by the walls or floor of the room (or objects within the room), the average irradiance at any given horizontal plane is roughly equivalent throughout the room. However, on a horizontal plane very close to the emitters, the irradiance on the plane is not uniform, with higher irradiances immediately below the emitter, and low or no irradiance in other locations. The installation is configured such that the location within the occupied zone, define as 2.1 m (7 ft) or less from the floor (depicted by the dashed line in Fig. 1), with the highest UV irradiance (designated with an “X”) complies with actinic ELs. Depending on the optical distribution of the emitters, this location may be directly below a single emitter or at a location where the radiation from two or more emitters overlaps significantly (as shown here). ELs may be exceeded only in the unoccupied space above 2.1 m (7 ft) *i.e.*, the upper room.

This article demonstrates analytically that the disinfection efficacy of a UV-DIBEL system at a given wavelength against a target pathogen is simply proportional to the allowed EL at that wavelength divided by the UV dose required for 90% inactivation; see Eq. (17). It is demonstrated herein that low-intensity UV-DIBEL irradiation can achieve high levels of equivalent air exchanges per hour (ACH_eq_) and can be an effective component of efforts to combat airborne pathogens such as SARS-CoV-2, Influenza A, the common cold, and others. The structure of the article follows these topics:

• the need for enhanced disinfection of indoor air to improve public health;
• the mechanisms responsible for indoor transmission of disease, focusing on airborne transmission;
• conventional mechanisms for inactivation of airborne pathogens;
• formalism for quantitative comparison of various air disinfection methods;
• safety standards that quantify the EL constraints of a DIBEL method;
• UV-C dose, *D*_90_, required for 90% inactivation of representative airborne pathogens;
• quantification of the disinfection efficacy of a DIBEL protocol in terms of EL/*D*_90_;
• quantitative examples of the disinfection efficacy of DIBEL protocols subject to ELs;
• anticipated evolution of DIBEL efficacy to ACH_eq_ > 100 h^−1^ with advanced optics and shorter-wavelength UV-C LEDs; and
• health hazards related to accidental exposure above the EL.

## 2 Background on Respiratory Diseases, Transmission, and Inactivation

### 2.1 Deaths from Major Infectious Diseases

Infectious diseases are among the top causes of death in the United States each year, including healthcare-associated infections (HAIs, ~70,000) and influenza and pneumonia (~50,000), totaling >100,000 per year. In addition, ~300,000 to 400,000 COVID-19 deaths occurred in both 2020 and 2021. With an unpredictable toll from COVID-19^3^3 COVID-19 refers to coronavirus disease 2019, which is caused by the severe acute respiratory syndrome coronavirus 2 (SARS-CoV-2). beyond 2021, we may expect at least 100,000 deaths per year from infectious diseases. Taken together, infectious diseases rank third behind only heart disease and cancer deaths in the United States [9]. Likewise, global deaths from tuberculosis alone totaled 1.4 million in 2019, and deaths from influenza A and pneumonia totaled 3.4 million [10]. Most of these infectious diseases having high death rates as a fraction of the population are transmitted by air or on surfaces following airborne flight. Each of these airborne transmission modes is susceptible to UV disinfection, such that widespread adoption of UV disinfection could have significant positive impact on public health.

### 2.2 Mechanisms of Disease Transmission Indoors

Airborne infections are transmitted primarily indoors. In contrast, airborne pathogens typically disperse rapidly in unconfined outdoor air and are usually not inhaled in a sufficient dose to be infectious. In the case of the initial strain of SARS-CoV-2, indoor transmission of disease is 18.7 times more likely than outdoor transmission, with similar trends reported for influenza and other respiratory diseases [11]. Many respiratory pathogens, including the SARS-CoV-2 virus, are transmitted via three principal mechanisms: inhaling infectious airborne aerosols (<100 μm diameter) [12, 13, 14], which may remain suspended in air for many seconds to hours, diffusing rapidly to fill the entire room; accruing infectious airborne droplets (>100 μm diameter) that have settled onto, or been inhaled by, a susceptible individual before they fall to the floor (within 1 m to 2 m) or are deposited onto surfaces, typically within a few minutes; and touching contaminated surfaces (fomites) before the pathogen decays (“dies”), typically in a few days. Additionally, unshielded coughs or sneezes can produce clouds that travel much farther than 1 m to 2 m [15, 16].

### 2.3 Inactivation Mechanisms for Airborne Pathogens

Most airborne infectious diseases are transmitted by vegetative bacteria, viruses, fungi, or bacterial spores [3]. These pathogens may be inactivated by any of several external stimuli, including UV radiation [3] and other external agents. Bacteria and fungi may be rendered unable to survive, reproduce, or infect a host and thus are inactivated. Viruses and bacterial spores have no metabolic processes but may be rendered unable to replicate in a host, and thus are also inactivated [3]. Pathogens may also be physically removed from air in the occupied environment, *e.g.*, by ventilation or filtration of the air. It is notable that, heretofore, most conventional air disinfection methods have physically removed airborne pathogens from the occupied zone to be inactivated or filtered in a separate zone that is protected from the occupied zone, *e.g.*, inside heating, ventilation, and air conditioning (HVAC) ducts, or in the upper room, or in an enclosed in-room cabinet. The germicidal UV (GUV) light source in an HVAC duct, or upper room UV germicidal irradiation (UR-UVGI) system [17, 18], or in-room UV enclosure may provide as much as 99.9% inactivation, or more, in the deactivation zone.

Ultraviolet light in the UV-A range (315 nm to 400 nm) efficaciously inactivates many types of vegetative bacteria, including some of the leading organisms responsible for HAIs, such as *Staphylococcus aureus* (*S. aureus*), *Enterococcus faecalis* (*E. faecalis*), and *Escherichia coli (E. coli*). The 1 log_10_ to 3 log_10_ inactivation of dried samples on surfaces has been reported for these organisms at doses below the EL at 365 nm [19, 20, 21]. The dose required in the UV-A range is typically ~10^4^ greater than that in the UV-C range, but the EL in the UV-A is correspondingly ~10^4^ higher than in the UV-C range, enabling the use of DIBEL in the UV-A as well as the UV-C parts of the spectrum. One of the primary pathways of inactivation is the creation of reactive oxygen species in the bacterial cell, which cause oxidative damage to the cell and inability to replicate or survive. UV-A may also provide lesser, but significant, inactivation of bacterial spores, fungi, and viruses. Similar, but lesser, inactivation of some pathogens is provided by visible light, for example, at 405 nm [22].

Ultraviolet light in the UV-B range (280 nm to 315 nm) inactivates many types of pathogens; however, the relatively high actinic weighting relative to the germicidal efficacy renders the UV-B range less useful below EL doses than either UV-A or UV-C [23].

Ultraviolet light in the UV-C range (100 nm to 280 nm) has photon energies that are nearly resonant with the absorption bands of deoxyribonucleic acid (DNA) and ribonucleic acid (RNA), enabling very effective inactivation of many types of viruses and bacterial spores, as well as many types of bacteria [3, 24, 25, 26]. One of the primary pathways of inactivation in the UV-C range is the breakage of thymine (in DNA with absorption peak at approximately 270 nm) or uracil (in RNA with absorption peak at approximately 254 nm) bonds in the base pairs of the nucleic acids, resulting in the creation of dimers in place of the original bases [3]. In the absence of repair mechanisms (which may occur in metabolic bacteria), a sufficient accumulation of dimers may render the virus inactivated, unable to produce viable replicates, and therefore noninfectious.

## 3 Overview of Conventional UV Disinfection Methods

### 3.1 For Pathogens on Surfaces or in Water or in Air

UV has been used for decades to disinfect surfaces, water, and air, typically using high-intensity discharge light sources such as mercury, xenon, or high-power excimer lamps. Given the high intensity of the light sources that enable high irradiance onto the target pathogen, inactivation of pathogens at levels exceeding 99% in seconds or minutes is typically achievable. However, such high intensity requires remote or shielded operation of the UV light source from human occupants. In high-intensity water and air disinfection systems, the fluid typically flows rapidly through a zone of high-irradiance germicidal UV with a dwell time in the UV zone sufficiently long to provide 85% to 99+ % inactivation of the target pathogen(s). In the case of air disinfection for occupied spaces, a shielded zone of UV may be provided inside an HVAC duct or inside an enclosed cabinet provided with air flow to and from the occupied space. A common embodiment having the intense UV located remote from the occupants is UR-UVGI [27]. In applications with room height less than about 3 m (about 9 ft), the intense UV is collimated into a tight beam restricted to the space above about 2.1 m (about 7 ft), with any hazardous leakage of UV below that level conforming to the allowed EL. With higher ceiling heights, the need for collimation may be relaxed.

### 3.2 Distinction between Continuous and Episodic Disinfection

An episodic inactivation protocol is one that is performed occasionally on an as-needed or scheduled basis. Examples may include:

• cleaning a patient room in a hospital before a new patient is admitted;
• cleaning an aircraft cabin interior between flights; and
• daily cleaning of a common area or restroom in a public facility.

The technologies typically employed in episodic cleaning protocols, all requiring operator training, include:

• wiping with cleaning chemicals, such as bleach, alcohol, H_2_O_2_, *etc*.;
• intense UV radiation from ceiling fixtures, UV robots, or UV towers; and/or
• fogging, *e.g.*, with H_2_O_2_, ozone, triethylene glycol (TEG), or another germicidal agent.

Episodic cleaning protocols typically provide at least 99% inactivation of the target pathogen(s) and typically require ~10 min to 60 min, during which the space must be unoccupied.

The space and/or surface is thereby typically rendered free of an infectious concentration of pathogens in the air and/or on surfaces that have been properly treated. Upon re-introduction of an infectious individual or contaminated object (*e.g.*, clothing, instruments, food service, *etc*.), the concentration of pathogens immediately begins to rebuild. For example, if an individual with a respiratory infection enters the space, the concentration of pathogens in the air will continually increase with each exhalation from the infectious individual. If an individual with contaminated hands enters the space, then each surface touched becomes recontaminated. The dynamics are presented in Sec. 4.3.

A continuous inactivation protocol, in contrast, operates without interruption, perhaps for many hours or all day, potentially including periods when the space is occupied. These protocols include HVAC, UR-UVGI, enclosed cabinets, and DIBEL.

## 4 Limitations of Conventional UV Air Disinfection Methods

The conventional air disinfection technologies include GUV in air-handler units (AHUs) such as a duct in an HVAC system, UR-UVGI, UV cabinets, *etc*. They are limited by the flow rate of contaminated air from the occupied zone into the enclosed high-UV disinfection zone and the flow rate of disinfected air returned to the occupied zone. For HVAC systems, GUV effectiveness depends on the recirculated air flow rate driven by mechanical blowers, and since GUV systems are often in series with mechanical filters, the air flow rate may be limited. However, UR-UVGI systems have demonstrated very high effective air change rates, without the limitation of series mechanical filters [17]. Nevertheless, an analysis of the impact of GUV in AHUs using the Wells-Riley model predicted that a typically sized system can reduce infection risk by as much as 50% [28] and that even low-powered systems sized for coil maintenance produce an air-quality benefit [29].

A common metric for air disinfection technologies is the air-exchange rate (AER) measured in air changes per hour (ACH) [30], defined as the total volume of air, *Q*, that flows into a room in 1 h (m^3^ h^−1^) divided by the room volume, *V* (m^3^):

***ACH=Q/V***

Such conventional air disinfection technologies have typical ACH_eq_ values of about 1 h^−1^ to 5 h^−1^. UR-UVGI systems have been reported with ACH_eq_ up to ~20 h^−1^ to 40 h^−1^ [17]. ACH values in typical indoor environments are summarized in Table 1 using the check values (not standards requirements) from the Informative Appendix L of the Ventilation Rate Check Table of the American Society of Heating, Refrigerating, and Air-Conditioning Engineers (ASHRAE) Standard 62.1-2019 [31] and conversion of cubic feet per minute therein to ACH here by assuming a 2.7 m (~9 ft) ceiling height. Row 1 is also included as a baseline for the typical air-exchange rate in a residence without forced air ventilation.

**Table 1:** ACH values in typical indoor environments.

Setting	ACH [h^−1^]
Home—walls, windows, doors leakage	0.5
Motel, hotel guest room	1
Office, conference room Retail sales Dental, urgent care	1 2 3
Classroom, daycare Restaurant, public auditorium Health club aerobics room	4 6 9

Note that ventilation accrues other benefits to air quality besides disinfection, including control of humidity, temperature, odors, dust and airborne particles, volatile organic compounds (VOCs), and concentrations of oxygen, CO_2_, *etc*. However, the ventilation rate, expressed as AER in units ACH, does not accurately quantify the effective clean air changes for disinfection. Rather, the uncontaminated ACH_eq_ is the important quantity for air disinfection. ACH_eq_ quantifies the ability of an environmental control to kill or inactivate an airborne microorganism at the same rate as mechanical ventilation removes the airborne microorganism from a room as measured in ACH [18]. In a UV air disinfection system, ACH_eq_ quantifies the germicidal efficacy using a decay model; see Eq. (3) below.

ACH_eq_ differs from the supply air change rate, AER in units ACH, in that AER may contain a significant fraction of only partially cleaned recirculated air. Thereby,

ACH_eq_=∊*Q/V

where ε is the fraction of recirculated air that is free of pathogens.

For example, consider a system providing 4 ACH of recirculated air through an HVAC system, with no pathogen-free outdoor air introduced. Suppose an in-duct filter having a typical minimum efficiency reporting value (MERV) of 13 removes 50% of infectious aerosol from the recirculated air. The pathogen-free air delivered is 0.5 × 4 ACH = 2 ACH_eq_. For the purposes of air disinfection, each of the values in Table 1 should be multiplied by ε in any given application. If the in-duct filter is a high-efficiency particulate air (HEPA) filter (MERV 17 or higher) or high-intensity UV, then ε ≅ 1.0. In many applications, a MERV 13 (50%) or lower filter may be used for which ε ≤ 0.5. Thereby, typical indoor settings are providing ACH_eq_ ~ 1–5.

### 4.1 Decay Model for Concentration of Pathogens in Air *vs*. Time

In the absence of mechanisms to introduce new pathogens into an indoor space, and assuming uniform spatial distribution of pathogens throughout the space (*i.e.*, well-mixed air), the concentration of pathogens in air will decay due to several different mechanisms, according to Refs. [32, 33, 34]:

**Table:** 

	$n\left(t\right)=n_{0}\;e^{-R\;*\;t}$	**(3)**

The pathogen removal rate, *R*, is given by

**Table:** 

	$R\;={ACH}_{eq}+\kappa +\lambda$	**(4)**

where

*t* = time (h),
*n* = virus concentration (quanta/m^3^), where a quantum is defined as the dose of airborne droplet nuclei required to infect a susceptible person,
*n*_0_ = initial virus quanta/m^3^ at *t* = 0,
ACH_eq_ = inactivation rate (h^−1^) from an air disinfection system, such as ventilation, filtration or UV inactivation
κ = natural viral inactivation rate = 0.63 h^−1^ for SARS-CoV-2 in still air at 25 °C [35], and
λ = deposition rate (h^−1^) onto surfaces due to gravitational settling and surface adsorption.

Per Eq. (3), if *R* = 1 h^−1^, then after *t* = 1 h, *n* is reduced to e^−1×1^ = 0.37 times the initial *n*_0_. Therefore, each unit of *R* produces a 63% reduction in airborne viral concentration per hour, regardless of whether the reduction is due to natural decay of the virus, or air exchange or UV inactivation, or settling to the floor and surfaces, or other mechanism. Note that one air change does not imply that 100% of the air in the space has been replaced; rather, it means that 1 − e^−1^ = 63% of the air in the space has been replaced, assuming a well-mixed space. After a time of 2 (3, 4) h, with *R* = 1 h^−1^, the airborne viral concentration is reduced by 86% (95%, 98%), and so on. In this formalism, ACH_eq_ contributes to the total virus removal rate, *R*, in the same way as the other virus-removal mechanisms, so that UV inactivation may be expressed by an ACH_eq_ value for direct comparison with the other virus-removal rates.

Further, Eq. (4) indicates that contributions to *R* are additive, such that ACH_eq_ from UV disinfection will be independently additive to disinfection by filtering or ventilation or other means. In this way, any addition to *R* from the ACH_eq_ of a UV disinfection system contributes to a multilayered infection-control strategy. For example, an AHU providing 3 ACH_eq_ supplemented by a UV-DIBEL system providing 3 ACH_eq_ provides a total 6 ACH_eq_ to the space.

The sum of natural decay, κ, and settling, λ, is ~1 ACH_eq_ for SARS-CoV-2. If a DIBEL system is designed to enhance the removal of pathogens significantly beyond the rate of natural removal mechanisms, then for simplicity, the natural removal mechanisms may be ignored as components of ACH_eq_. When natural decay and settling are ignored, Eq. (3) becomes

**Table:** 

	$n\left(t\right)=n_{0}\;e^{-{ACH}_{eq}\;*\;t}$	**(5)**

### 4.2 Inactivation Rates for Viruses in Air Irradiated with UV-C

The UV dose required for 90% inactivation, *D*_90_, has units of joules per square meter (J/m^2^) (sometimes expressed in μJ/cm^2^ or mJ/cm^2^). For UV disinfection of air, the infectious pathogen inactivation rate, *R*, in Eq. (4), which equals ACH_eq_ in Eq. (5), is often quantified using *Z* (m^2^/J), the UV susceptibility constant for the pathogen, where *R* = ACH_eq_ = *Z* × *E*, and *E* is the irradiance (W/m^2^) [36]. *Z* is also sometimes referred to as the UV rate constant, *k*, (m^2^/J) [3]. *Z* and *k* are related to *D*_90_ by [3]:

**Table:** 

	$Z\;=k\;=\frac{2.30}{D_{90}}\;or\;D_{90}=\frac{2.30}{Z}$	**(6)**

The time (h) for a *D*_90_ dose to be delivered (*t*_90_) at a continuous, uniform irradiance, *E* (W/m^2^), into the deactivation zone is:

**Table:** 

	$t_{90}=D_{90}/\left(3600*E\;\right)$	**(7)**

In conventional air-moving UV disinfection systems (AHUs, UR-UVGI, cabinets), the *t*_90_ value inside the high-intensity UV zone is typically only a few seconds, but the overall ACH_eq_ delivered to the occupied zone is limited by the much slower air-exchange rate of the fans moving air through the disinfection zone. In such systems, ACH_eq_ cannot exceed the system air-flow rate. UR-UVGI systems can achieve high ACH_eq_, but only if there is good air mixing in the space that moves air through the disinfection zone. In contrast, air disinfection in a DIBEL system does not require air movement, because the pathogens are inactivated in the occupied zone. Using Eq. (5), the ACH_eq_ for a DIBEL system may be directly compared with the ACH_eq_ of conventional air-moving UV disinfection systems (Table 1), which are limited by the air-flow rate through the disinfection zone.

For a DIBEL system, neglecting the pathogen removal due to natural decay, settling, and air movement, evaluation of Eq. (5) at *t* = *t*_90_ provides

**Table:** 

	$\frac{n\left(t=t_{90}\right)}{n_{0}}=0.1=\;e^{-{ACH}_{eq\;}*\;t_{90}}$	**(8)**

Solving Eq. (8) for ACH_eq_ for a DIBEL system gives:

**Table:** 

	${ACH}_{eq}=\frac{\ln\left(10\right)}{t_{90}}=2.30*3600*E/D_{90}$	**(9)**

From Eq. (9), the ACH_eq_ value for DIBEL is inversely proportional to the *D*_90_ value of the pathogen. Therefore, the ACH_eq_ for a DIBEL system must be stated relative to the target pathogen.

Using values for SARS-CoV-2, the natural decay of aerosolized virus is 0.63 h^−1^, and the deposition rate to floor and surfaces in typical-sized rooms for exhaled aerosolized particles is ~0.3 h^−1^ [32], so the baseline value of *R* for SARS-CoV-2 is ~1 h^−1^. Therefore, any UV air disinfection method having

ACH_eq_ ~ 1 or more will significantly accelerate the exponential removal of viral quanta from the air and will be at least somewhat efficacious against SARS-CoV-2.

**Fig. 2:**
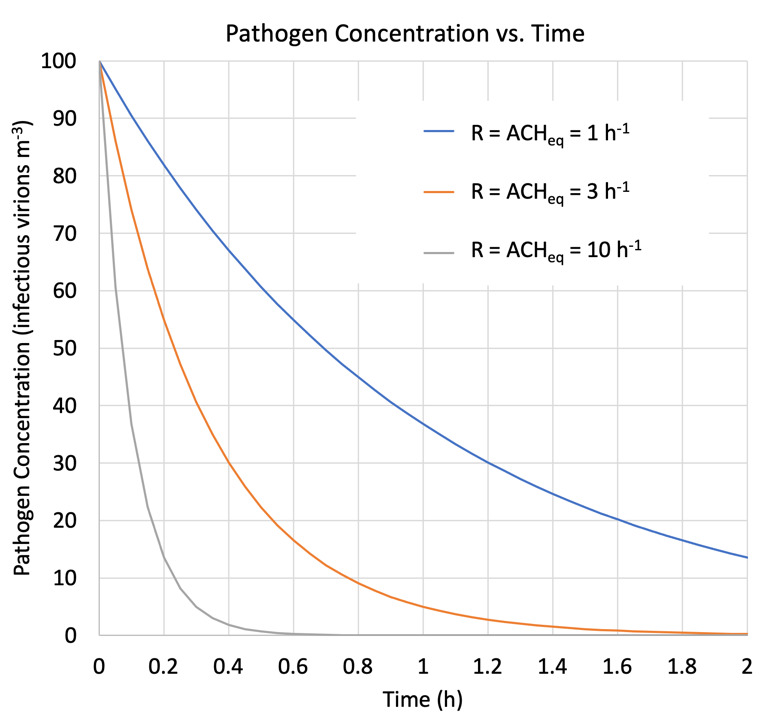
Concentration of airborne pathogens *vs*. time for *R* = 1, 3, and 10 h^−1^.

Figure 2 shows inactivation or removal of an initial concentration of infectious aerosol particles predicted by Eq. (5) assuming an initial pathogen concentration normalized to 100% for three exemplary cases: natural decay and gravitational settling only (ACH_eq_ = 1 h^−1^); a typical HVAC system in an office or residence (ACH_eq_ = 3 h^−1^); and a medical facility or other well-ventilated space (ACH_eq_ = 10 h^−1^).

On the timescale of interest (<1 h) for eliminating pathogens from the air to reduce the risk of airborne disease transmission, Fig. 2 demonstrates the potentially very significant impact of actively increasing ACH_eq_ beyond that of the passive natural decay and settling of an airborne pathogen.

### 4.3 Model for Dynamic Pathogen Removal *vs*. Time

In contrast to Eq. (3), which applies in the absence of mechanisms to introduce pathogens into an indoor air space, Eq. (10) includes a source of pathogens introduced into an indoor space by exhalation from an infectious subject [32]:

**Table:** 

	$n\left(t\right)=\frac{R_{e}*N}{R*V}+\left(n_{0}-\frac{R_{e}*N}{R*V}\right)*e^{-R*t}$	**(10)**

where

*R*_e_ = emission rate (h^−1^) of pathogens exhaled per hour per infectious subject,
*N* = number of infectious individuals in the space, and
*V* = volume of the space (m^−3^).
At *t* = ∞,

**Table:** 

	$n_{\infty }=\frac{R_{e}*N}{R*V}$	**(11)**

Therefore, the steady-state density of pathogens scales as 1/*R*, which is the total pathogen-removal rate. Note that the steady-state pathogen density in the air,$\;n_{\infty }$, is also proportional to the number of infectors, *N*, multiplied by the emission rate, *R*_e_, of each infectious individual.

Equation (10) then simplifies to

**Table:** 

	$n=\left(n_{0}-n_{\infty }\right)e^{-R*t}+n_{\infty }$	**(12)**

**Fig. 3:**
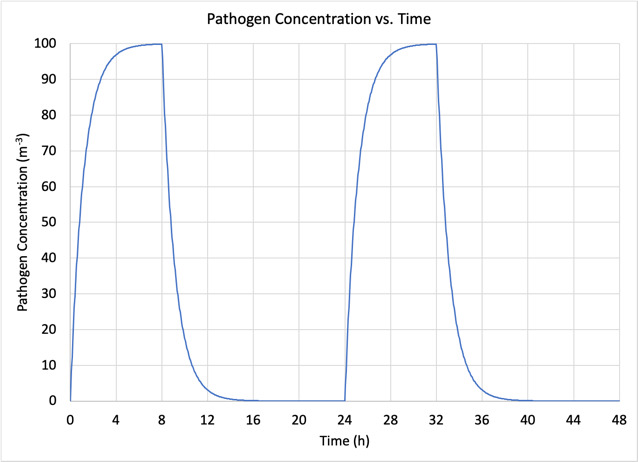

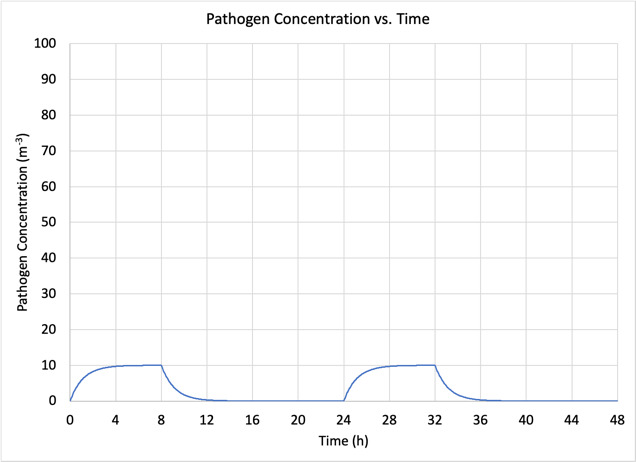
Concentration of airborne pathogens *vs*. time for baseline case without (a) and with (b) air disinfection.

Figure 3 (a and b) demonstrates the advantage of implementing a continuous air disinfection system, characterized by ACH_eq_ = 10 h^−1^ (Fig. 3[b]), relative to having no air disinfection and relying on natural decay and gravitational settling of the airborne pathogens, characterized by ACH_eq_ = 1 h^−1^ (Fig. 3[a]), with baseline conditions:

• *n*_0_ = 0,
• a single infectious individual (*N*=1) who enters the space for 8 h at the beginning of each day at *t* = 0, 24 h,
• an emission rate, *E* = 4000 h^−1^ (this is an arbitrary value, for arithmetic simplicity), and
• an indoor volume, *V* = 40 m^3^ (*e.g.*, a room approximately 4 × 4 × 2.5 m high).

For ACH_eq_ = 10 h^−1^, Eq. (11) becomes

**Table:** 

	$n_{\infty }=\frac{E*N}{R*V}=\frac{4000\;\left(h^{-1}\right)*1}{10\;\left(h^{-1}\right)*40\;\left(m^{3}\right)}=10\;\left(m^{-3}\right)$	**(13a)**

For ACH_eq_ = 1 h^−1^, Eq. (11) becomes

**Table:** 

	$n_{\infty }=\frac{E*N}{R*V}=\frac{4000\;\left(h^{-1}\right)*1}{1\;\left(h^{-1}\right)*40\;\left(m^{3}\right)}=100\;\left(m^{-3}\right)$	**(13b)**

Note that pathogen concentrations (*e.g.*, the number of infectious virions per cubic meter) of 10 m^−3^ and 100 m^−3^ in Eq. (13a) and Eq. (13b) are also arbitrary values, selected to simplify the demonstration of the relative reduction provided as a function of ACH_eq_.

For the case of $n_{0}=0$ Eq. (12) can be rewritten as

**Table:** 

	$n=\left(n_{0}-n_{\infty }\right)e^{-R*t}+n_{\infty }=n_{\infty }\left(1-e^{-R\;*\;t}\right)$	**(14)**

whereby the pathogen concentration in the air increases asymptotically from *n*_0_ = 0 to $n_{\infty }\;$in competition between the pathogen exhalation rate, *R*_e_, and the pathogen removal rate, *R*, as shown in Fig. 3. Figure 3(a) presents the special case of a pathogen removal rate *R* = 1 h^−1^ (natural decay rate of pathogen plus gravitational settling, without any ventilation or intentional disinfection). Figure 3(b) presents the special case of a pathogen removal rate *R* = 10 h^−1^ (provided by an air disinfection system, such as DIBEL).

The significance of Fig. 3 is that the 10× higher *R* values provided by the air disinfection system in Fig. 3(b) vs. that in Fig. 3(a) reduces the concentration of airborne pathogens by 10× at all times greater than zero, including the asymptotic equilibrium concentration. The impact on the probability of infection of a susceptible individual exposed to an individual infected with SARS-CoV-2 in typical indoor scenarios has been quantified from Monte Carlo calculations of a stochastic exponential dose–response model for airborne transmission of pathogens [32]. From Table 3 of Ref. [32], it can be inferred that an increase in ACH_eq_ from 1 h^−1^ to 10 h^−1^ would reduce the probability of infection over a 3 h period from a 10% risk to about a 2% risk. The assumptions and caveats accompanying that inference are extensive, but the 5× reduction in risk may be understood as an order of magnitude estimate.

## 5 Quantification of the Efficacy of UV-DIBEL Air Disinfection Methods

The benefits of a DIBEL technology that differentiate it from conventional disinfection technologies include:

• continuous disinfection while occupied,
• direct irradiation while occupied, and
• no required air movement, so that disinfection occurs in the space between an infected person and susceptible people, providing an effective shield between infectious and susceptible individuals that is proportional to the ACH_eq_ provided by the DIBEL system.

While the relatively poor electrical efficiency (presently <10%), low optical output (<100 mW), and high cost of UV-C LEDs generally limit their practical applications, these attributes are not disabling factors in DIBEL applications subject to the actinic EL (~1 mW/m^2^). In fact, the low optical output makes a typical UV-C LED today a nearly ideal light source to provide irradiance comparable to the actinic EL on a plane 2.1 m above the floor from a typical 2.4 m to 2.7 m- (8 ft or 9 ft) high ceiling. As such, UV-C LEDs, having near-Lambertian light distribution, may be spaced apart by 1 m to a few meters in an array at the ceiling to provide acceptably uniform irradiance in the occupied zone without the need for special optics. However, with beam-forming optics to provide near-field uniformity, the spacing between LED emitters in the array may be increased several-fold, with the advantages of both lower acquisition cost for the DIBEL hardware and more uniform irradiance throughout the occupied space.

Since DIBEL protocols are limited by EL, which has units of joules per square meter (J/m^2^), it is convenient to express the UV irradiance (W/m^2^) in terms of UV fluence, *F*, also having units joules per square meter (J/m^2^) [37], by multiplying the UV irradiance by the exposure time, *T* (s), and converting seconds to hours, since the typical *t*_90_ inactivation times for DIBEL are ~1 h. (Note that the term “fluence” as used herein means the time-integrated irradiance incident on a planar surface, which should not be confused with alternate uses of the term found in the literature, such as the time-integrated irradiance incident on a spherical surface, which will be defined herein as “spherical irradiance”.)

Thus:

**Table:** 

	$F\;=E\;*T\;\left(s\right)\;=\;3600*E\;*T\;\left(h\right)$	**(15)**

Equations (7) and (9) may be rewritten as follows, where the UV fluence, *F*, is a fraction, *f* ≤ 1 (an engineering margin), of the maximum allowed fluence, EL.

**Table:** 

	$t_{90}=D_{90}/\left(3600*E\right)=\frac{D_{90\;}\;*\;T\;\left(h\right)}{f*EL}$	**(16)**
	${ACH}_{eq}=\frac{\ln\left(10\right)}{t_{90}}=2.30\times 3600\times \frac{E}{D_{90}}=\frac{2.30\;*\;f*EL}{D_{90\;}*T\left(h\right)}$	(17)

Substituting the maximum allowed value of *F* = EL (*f* = 1.0) into Equations (16) and (17) quantifies the theoretical potential capability of a DIBEL system for air disinfection in terms of EL, *D*_90_, and the exposure time, *T*.

**Table:** 

	$t_{90}=\frac{D_{90\;}*\;T\;\left(h\right)}{EL}$	**(18)**
	${ACH}_{eq}=\frac{2.30\;*\;EL}{D_{90\;}*T\left(h\right)}$	(19)

### 5.1 Quantifying the Actinic Hazard Limit

**Fig. 4:**
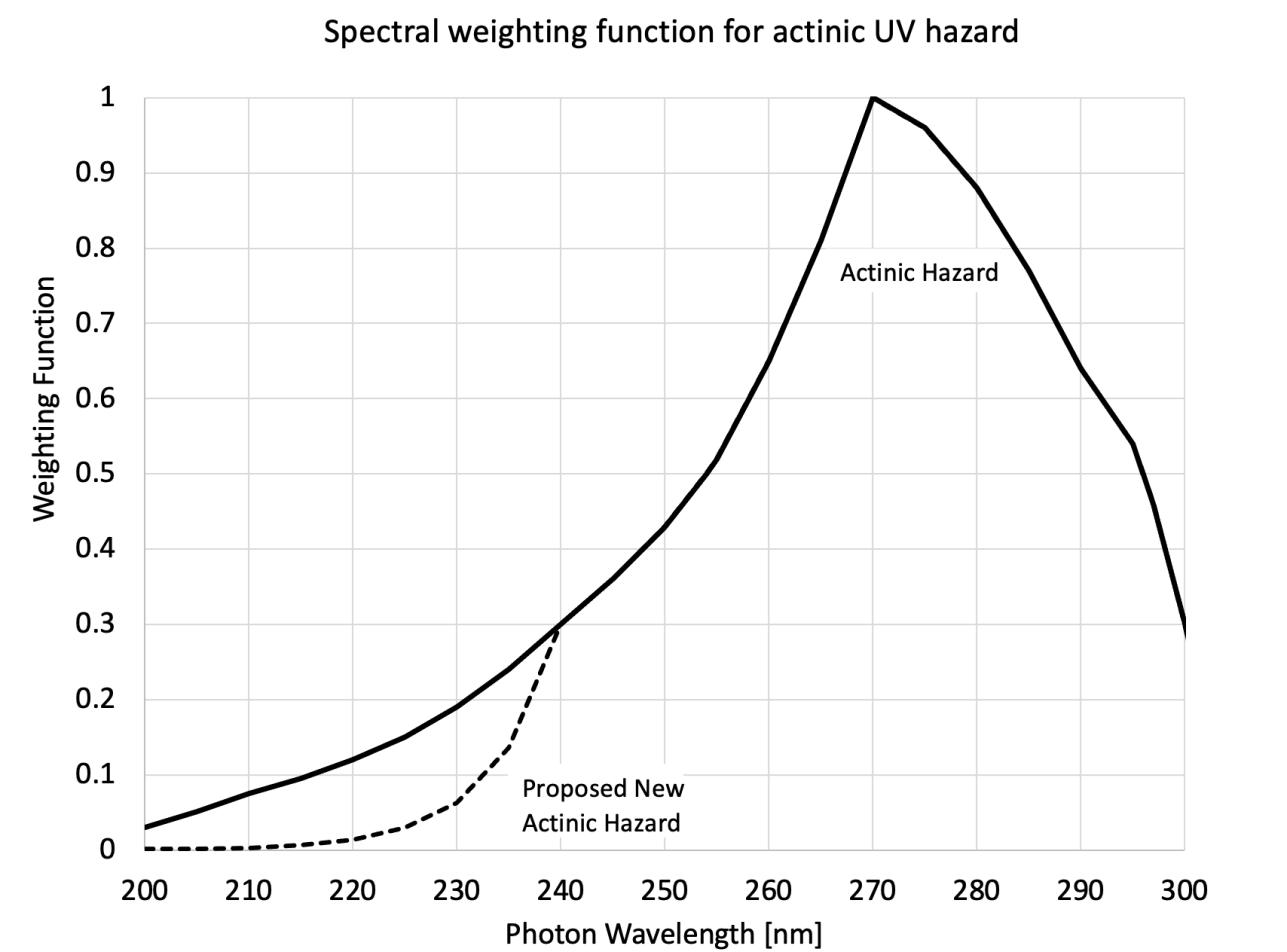
Spectral weighting function for actinic UV hazard *vs*. wavelength from IEC 62471:2006 [5].

Of the several categories of photobiological hazards, only the actinic hazard pertains to the UV range. The EL for actinic hazard as provided in International Electrotechnical Commission (IEC) Standard 62471:2006 [5] is 30 J/m^2^ in any 8 h period. The actinic hazard function shown in Fig. 4 is normalized to 1.0 at its peak of 270 nm. Shorter and longer wavelengths, being less hazardous, are assigned weighting factors <1.0, which are divided into 30 J/m^2^ to obtain the EL at each corresponding wavelength. The dashed curve in Fig. 4 at wavelengths below 240 nm pertains to a proposed change to the ELs below 240 nm at some future date. For example, the weighting and allowed dose for UV-C wavelengths of practical interest for viral inactivation are listed in Table 2.

EL values from Fig. 4 representing present and potential future DIBEL applications with UV-C LEDs are shown in Table 2.

**Table 2:** EL values of particular interest for DIBEL with UV-C LEDs.

**Wavelength (nm)**	**Actinic Weight**	**EL (J/m^2^)**
**275**	0.96	31
**255**	0.52	58
**235**	0.24	125
**225**	0.15	200

From Eq. (17), ACH_eq_ may be increased by increasing *F* (up to the EL limit) and decreasing the exposure time, *T*, for a given pathogen having a given *D*_90_. For example, with *F* = EL = 31 J/m^2^ (at 275 nm) and *D*_90_ = 5 J/m^2^ (*e.g.*, for SARS-CoV-2), then

**Table:** 

	${ACH}_{eq}\;=\frac{2.30\;*\;31}{5\;*\;T\;\left(h\right)}=\frac{14}{T\;\left(h\right)}$	**(20)**

ACH_eq_ for SARS-CoV-2 in air at 275 nm could theoretically be as high as about 2 h^−1^ for a continuous 8 h exposure at the EL, or as high as about 14 h^−1^ for a continuous 1 h exposure at the EL.

### 5.2 Attributes of a Typical DIBEL Protocol

The term “DIBEL protocol” is used to mean the application of a DIBEL system operating with the following attributes specified:

• wavelength (or band or range of wavelengths);
• spatially averaged irradiance in the occupied space;
• exposure time, or irradiance schedule over time;
• optical distribution (*e.g.*, targeting air volume or surfaces, either the entire space or a subset); and
• target pathogen(s) and medium.

The fraction, *f*, of the maximum allowed value of *E* throughout the irradiated space relative to EL/*T* may be estimated as follows. In practice, the irradiance will be nonuniform, with “hot spots” that may not exceed EL at any location within the occupied zone, and with “cold spots” due to shadows and beam pattern of the UV sources, *etc*., such that a practical uniformity achieved by optimizing the layout and optical distribution of the UV light sources (akin to “illuminating engineering” for visible lighting) provides *E*_avg_/*E*_max_ ~ 80%. Furthermore, a conservatively designed DIBEL system should not result in *E*_max_/EL greater than ~ 80%, allowing for an “engineering margin.” Thus *f* = 0.64 may be estimated as practical value.

For example, a typical DIBEL protocol might comprise:

• an LED with peak wavelength at 275 nm;
• nearly uniform (80%) irradiance throughout the occupied space;
• *F*_max_ = *E*_max_ × *T* = 0.8 × EL, providing a 20% engineering margin relative to EL;
• an average spatially variable fluence, *F*_avg_, of 20 J/m^2^ (64% of the EL = 31 J/m^2^ at 275 nm), which is integrated throughout the occupied volume;
• continuous, constant irradiation for *T* = 8 h;
• *E* = 20 J/m^2^/8 h = 2.5 J/m^2^-h = 0.7 mW/m^2^;
• a target pathogen specified as SARS-CoV-2 virus in air, for which *D*_90_ ~ 5 J/m^2^;
• an ACH_eq_ = 1.1 h^−1^, from Eq. (17);
• a single 275 nm LED covering an ~10 m^2^ floor area in the irradiated space;
• 7 mW of UV output from the LED (commercially available with >10,000 h life);
• 0.1 W electrical input at 7% wall-plug efficiency (WPE), consuming only ~0.01 W/m^2^ of electrical load per floor area (<1% of a typical lighting load); and
• a ceiling-mounted installation in a small, unobtrusive housing requiring no heat sink.

A comparison of the DIBEL result of ACH_eq_ = 1.1 h^−1^ in the example protocol above with the typical values for conventional air disinfection in Table 1 indicates that certain DIBEL protocols may provide comparable ACH_eq_ to the lower range of conventional air disinfection methods in residential and commercial settings (~1 ACH_eq_ to 5 ACH_eq_). A DIBEL protocol that incorporates just one or a few UV LEDs, mounted in the ceiling of the occupied space, in an unobtrusive housing similar in appearance to a smoke detector or small lighting fixture, may provide significant benefits relative to the considerable infrastructure modifications required to install or upgrade conventional air disinfection systems. As such, DIBEL with today’s UV-C LEDs may be considered in a layered strategy for air disinfection, complementing existing systems as a relatively convenient and inexpensive upgrade, and in addition to other infection control protocols such as surface cleaning, hand washing, and mask wearing. However, as shown in Sec. 5.3, the near-term evolution of DIBEL with UV-C LEDs may be expected to achieve parity with, and then exceed, the germicidal efficacy of conventional air disinfection systems. Later sections will quantify how the typical ACH_eq_ of 1.1 h^−1^ may be enhanced by 1 to 2 orders of magnitude as DIBEL technologies evolve.

### 5.3 Anticipated Increased Efficacy of DIBEL with Evolution of UV-C LEDs

UV-C LEDs having a peak wavelength at 275 nm are readily available commercially with the following favorable attributes:

• LED cost low enough to be a minor cost in a DIBEL system,
• WPE of ~1% to 5%, and improving, such that electrical load is <<0.1 W/m^2^ of treated area, and
• lifetimes ~2000 h to 10,000 h and improving.

Comparable attributes to those listed above for 275 nm LEDs are evolving commercially now for 265 nm LEDs and soon emerging for 255 nm LEDs. Sample LEDs are already available at 235 nm, with the shortest laboratory demonstration at 210 nm, very close to the band edge (theoretical minimum wavelength) of aluminum nitride (205 nm) [7].

Although excimer discharge lamps already provide sufficient UV output and WPE at 222 nm for reasonable cost/performance as an entry DIBEL protocol [38], they are a mature technology, without the promise of significant reduction in cost or size or increase in life with further development. They are, and will probably remain, relatively expensive and large light sources, with relatively short lifetimes compared with the near-term expected lifetimes of UV-C LEDs. If, as expected, UV-C LEDs migrate to wavelengths <250 nm, and then <230 nm, they will become preferred over excimer light sources based on the favorable attributes of UV-C LEDs, including cost, size, WPE, and optical beam control, which is limited by the light source size (etendue).

UV-C LED WPE has only recently risen to ~10%, but anticipated improvements in epitaxial structures are predicted by some to enable WPE improvements to 30% by 2025 and 50% by 2030, following the historical trajectory of WPE for blue LEDs assuming a 25 year lag time and a gentler slope [7]. Even if the forecast WPE trajectory is optimistic, UV-C DIBEL systems are already commercially viable with WPE ~5%, consuming <<0.1 W/m^2^, and they will benefit from even modest WPE improvements.

Therefore, as depicted in Table 3, it is reasonable to estimate the capabilities of UV-C LED DIBEL protocols into the near future [7] based on the minimum wavelength of commercially available LEDs *vs*. year, assuming LED lifetime of at least 10,000 h and WPE of at least 1%. In Table 3, the engineering margin of *f* = 0.64 (*F* = 0.64 × EL) has been incorporated into the calculation of ACH_eq_, in Eq. (17), with *D*_90_ = 5 J/m^2^ and *T* = 8 h. It is assumed here that *D*_90_ = 5 J/m^2^ for SARS-CoV-2 in air, independent of wavelength in the UV-C. While this may be approximately true, it has not yet been experimentally verified for SARS-CoV-2 in air, although *D*_90_ for MS2 (a recognized surrogate for coronaviruses) has been demonstrated to vary by <3× over the range 200 to 275 nm [39]. No optical enhancement of the fluence is assumed; this is addressed in Sec. 6.

**Table 3:** Impact of anticipated emergence of UV-C LEDs for DIBEL applications.

**Anticipated LED Availability Date**	**Minimum LED Wavelength Commercially Available (nm)**	**Actinic Weight**	**EL (J/m^2^)**	**Proposed Future EL (J/m^2^)**	**ACH_eq_ (h^−1^) from Eq. (17)**
**2020**	275	0.96	31		1.1
**2025**	255	0.52	58		2.1
**2030**	235	0.24	125	216	~5 to 8
**2035**	225	0.15	200	1012	~7 to 40

The “Proposed EL” column, taken from the dashed curve in Fig. 4, represents a change to EL in the actinic hazard standard that was recently proposed for wavelengths below 240 nm, with recommended implementation into standards in the near term. The proposed change recognizes the degree to which wavelengths <240 nm are attenuated at the uppermost layers of human skin and eyes, without significantly penetrating to the living cells below.

Table 3 suggests that DIBEL systems incorporating UV-C LEDs will improve considerably from their present relative parity with conventional air disinfection methods in germicidal efficacy to eventually exceeding conventional systems, when operated continuously for 8 h periods in an occupied space. The ACH_eq_ values in Table 3 estimated using Eq. (17) for 225 nm LEDs, along with the proposed increased EL shown in Fig. 4, indicate a trajectory for DIBEL with UV-C LEDs to achieve ACH_eq_ ~ 50 h^−1^ for a continuous 8 h exposure, without optically enhanced fluence.

Furthermore, a DIBEL system may be operated continuously for a period less than 8 h, *e.g.*, for an hour in a conference room, with the entire 8 h EL dose being irradiated in 1 h, thereby boosting the ACH_eq_ values listed in Table 3 by 8×, extending up to 400 h^−1^, as long as system controls limit the exposure of any one individual to only one such exposure in the permitted time period. Other DIBEL protocols incorporating occupancy and/or proximity sensors, timers, and system-integrated controls may further enhance ACH_eq_ in challenging or especially hazardous situations.

### 5.4 Example *D*_90_ Values of Pathogens in Air

A summary of the dose of 254 nm UV-C required for 90% inactivation (*D*_90_) of viruses in air from the full database provided in Kowalski [3] is shown in Table 4.

**Table 4:** Summary of all dose data at 254 nm UV-C for 90% inactivation (D_90_) of viruses in air taken from the full database provided in Kowalski [3].

**Virus**	***D*_90_ (J/m^2^)**
**Adenovirus**	44
**Bacteriophage MS2**	12
**Coliphage T7**	8
**Coliphage fX-174**	3
**Coronavirus**	6
**Coxsackievirus**	21
**Influenza A**	19
**Phage phi 6**	6
**Sindbis virus**	22
**Vaccinia virus**	4
**Geometric mean**	10

(Note, the *D*_90_ value for coronavirus reported in Walker and Ko [40] is 6 J/m^2^, differing from 3 J/m^2^ reported in Kowalski [3].) The coronavirus shown here is murine hepatitis virus, not to be confused with SARS-CoV-2.

A conclusion from Table 4 is that a typical value for *D*_90_ for viruses in air at 254 nm is 10 J/m^2^.

### 5.5 *D*_90_ for SARS-CoV-2 in Air

Several reports have been published or announced online for *D*_90_ measurements of SARS-CoV-2 in aqueous solutions and on surfaces, but none yet for aerosol. Beggs and Avital [36] assimilated many such *D*_90_ reports and estimated that *D*_90_ in air for SARS-CoV-2 is likely to be in the range *Z* = 0.377 to 0.590 m^2^/J. From Eq. (6), *D*_90_ = 3.9 to 6.1 J/m^2^. A value of *D*_90_ ~ 5 J/m^2^ will be assumed for SARS-CoV-2 in air at 254 nm herein.

## 6 DIBEL Efficacy Against Various Airborne Pathogens

The *D*_90_ values in air at 254 nm for viruses, bacteria, spores, and fungi of interest in public health are listed from Kowalski [3], except for SARS-CoV-2 derived herein, in Table 5. Where Kowalski provided multiple *D*_90_ values for a given pathogen in air, the geometric mean value is listed here.

**Table 5:** *D*_90_ values in air for viruses, bacteria, spores, and fungi of interest in public health.

**Pathogen**	**Type**	***D*_90_ in Air (J/m^2^)**	***D*_90_ Category**
**SARS-CoV-2**	Virus	5^a^	Low
***Mycobacterium tuberculosis***	Bacteria	5	Low
***Staphylococcus aureus* (*e.g.*, Methicillin-resistant *Staphylococcus* aureus, MRSA)**	Bacteria	5	Low
**Coronavirus (some common colds)**	Virus	6^b^	Low
**Pathogens responsible for pneumonia: *S. aureus* (5), *Klebsiella pneumoniae* (7), *Pseudomonas aeruginosa* (4), *Streptococcus pneumoniae* (~5)**	Bacteria	6^c^	Low
***Escherichia coli***	Bacteria	8	Low
**Influenza A**	Virus	19	Medium
**Adenovirus**	Virus	44	High
***Candida auris***	Fungus	~50^d^	High
***Clostridioides difficile***	Bacterial spore	~50^d^	High

^a^ SARS-CoV-2 estimated from compilation of data at various wavelengths on various media [36].

^b^ From Walker and Ko [40].

^c^ Geometric average of *D*_90_ from several bacteria responsible for pneumonia.

^d^ Approximated from *D*_90_ for similar pathogens in water and on surfaces, with corrections [3] for air.

From Table 5, it may be inferred that airborne pathogens may be categorized for convenience by three representative values of *D*_90_: low ~ 5 J/m^2^, medium ~ 20 J/m^2^, and high ~ 50 J/m^2^. With that, ACH_eq_ may be estimated for the minimum available wavelength of UV-C LED in 2020–2021, and for future application, for an 8 h continuous DIBEL protocol, as shown in Table 6, again incorporating the *f* = 0.64 engineering factor of Eq. (17).

**Table 6:** Estimated evolution in ACH_eq_ over time based on the minimum available wavelength of UV-C LEDs and improved optical distribution for an 8 h continuous DIBEL protocol for the low, medium, and high categories of pathogens based on *D*_90_ in air at 254 nm.

*D*_90_ Category in Air (J/m^2^)	Example Pathogens	ACH_eq_ (h^−1^)			
		2020 (275 nm)	2021 (275 nm + optics)	2025 (255 nm + optics)	Potential (225 nm + optics)
Low ~ 5	SARS-CoV-2, tuberculosis, pneumonia-causing bacteria, MRSA	1.1	4	8	150
Medium ~ 20	Influenza A	0.3	1.2	2.2	40
High ~ 50	Adenovirus, *C. auris, C. difficile*	0.1	0.4	0.8	15

In the 2021 and later columns of Table 6, an improvement of ~4× in ACH_eq_ is included, which has been realized in practice from adjustment of the optical distribution throughout the irradiated space, denoted by “+ optics” in the column headings. This improvement derives from an enhancement of the volume-averaged spherical irradiance relative to the planar irradiance at 2.1 m above the floor, which determines EL. The enhancement is made possible [41] by consideration of the geometric difference between the spherical irradiance incident on an airborne pathogen compared with the planar irradiance measurement specified in the actinic hazard standard [5] representing the incident planar irradiance on the eye or the skin.

The significance of Table 6 is that the long-term potential for DIBEL in air with UV-C LEDs is ACH_eq_ ~ 150 h^−1^ for the most susceptible pathogens and ACH_eq_ ~ 40 h^−1^ for most other pathogens for continuous irradiation over an 8 h period, exceeding the capabilities of conventional air disinfection technologies.

## 7 Safety of DIBEL Disinfection Protocols

### 7.1 Wavelength Ranges of Commercially Available DIBEL Products

There are currently four wavelength bands represented by commercial products providing DIBEL disinfection of air and/or surfaces in occupied spaces as follows:

• visible (400 nm to 700 nm) 405 nm to 420 nm LEDs,
• UV-A (315 nm to 400 nm) 365 nm LEDs,
• UV-C (100 nm to 280 nm) 255 nm to 280 nm LEDs, and
• far UV-C (nominally < 240 nm) 222 nm excimer.

The latter two categories may eventually merge as commercially available UV LEDs evolve to shorter wavelengths. The UV-B band (280 nm to 315 nm) is generally not favorable for DIBEL applications, because the EL values are generally low relative to *D*_90_ for pathogens of interest in this range. The EL increases relatively slowly *vs*. wavelength between 280 nm and 315 nm [5], while *D*_90_ values typically rise abruptly beyond 280 nm.

### 7.2 Safe Use of UV Light

Every wavelength of light, from the UV through the visible and into the infrared (IR), poses a health risk to humans if the dose of light exceeds the allowed EL. UV is no more hazardous than visible or IR light when the dose is maintained below the allowed EL. Conversely, when received at a dose exceeding the EL for visible light, visible light is more hazardous than UV light maintained below its respective EL.

UV-C is no more dangerous than visible or UV-A or far UV-C light when the dose is maintained below the EL. Even though the EL varies by about 10,000 times through the UV range, the germicidal efficacy against many target pathogens also varies, by about the same 10,000 times, such that many wavelengths in the UV-C, UV-A, and far UV-C ranges may each be applied safely with germicidal efficacy when the daily dose to humans in the irradiated space remains below the EL corresponding to the wavelength of light.

#### 7.2.1 Safety Standards for UV Radiation

Every DIBEL protocol involves ensuring that the dose (irradiance × exposure time) received by any individual at any location in the irradiated space remains below the EL corresponding to the wavelength(s) of the light. The EL values for all wavelengths of light from 200 nm through 3000 nm are provided in several standards documents:

• IEC Standard 62471-1:2006 [5] and Commission International de l’Eclairage (CIE) Standard S-009E-2002 [42], Photobiological Safety of Lamps and Lamp Systems;
• International Commission on Non-Ionizing Radiation Protection (ICNIRP), Guidelines on Limits of Exposure to Ultraviolet Radiation of Wavelengths between 180 nm and 400 nm (Incoherent Optical Radiation) [43];
• ACGIH^®^, Threshold Limit Values (TLVs^®^) and Biological Exposure Indices (BEIs^®^) [44]; and
• American National Standards Institute and Illuminating Engineering Society (ANSI/IESNA) RP-27, Photobiological Safety for Lighting Systems [45].

The limits defined by these standards represent the conditions to which nearly all individuals can be repeatedly exposed for 8 h per day over a working lifetime without risk of photobiological effects such as skin or eye damage or irritation. The various wavelength ranges of present consideration in DIBEL applications, namely, 222 nm, 254 nm, 365 nm, and 405 nm, are subject to three different hazard functions, following the terminology used in IEC 62471-1:2006 [5], listed here:

• 200–400 nm actinic hazard, which applies to 222 nm, 255 nm to 280 nm, and 365 nm;
• 315–400 nm near-UV hazard, which applies to 365 nm; and
• 300–700 nm retinal blue light hazard, which applies to 405 nm.

Although a single wavelength is usually used to describe the emission from an LED or excimer light source, it is important to note that the EL values derived from these hazard functions must take into consideration the full spectral power distribution (SPD) of the light output *vs*. wavelength of the source being evaluated. A typical UV LED has a near-Gaussian SPD characterized by a full width at half maximum (FWHM) of ~10 nm; a typical excimer lamp SPD has one or more lines in addition to a long-wavelength band extending over tens of nanometers. When the EL is calculated as an integral over the full SPD of a typical UV LED, as required in the IEC standard, the resulting EL differs by 5% or less relative to simply evaluating the EL at the peak wavelength of the LED. If an excimer light source is filtered against the long-wavelength band, a similar result would hold. Therefore, it is convenient and sufficiently precise to evaluate the EL of a UV LED or a properly filtered excimer lamp at the peak wavelength of the light source, without evaluating the exact integral over the SPD.

#### 7.2.2 Hazards of UV Light Above the Exposure Limits

Although DIBEL protocols may be engineered to be essentially risk-free with careful optical design, sensors, and controls, the impact of potential overexposure should nonetheless be considered [46, 47]. The hazard function pertaining to the range 200 nm to 400 nm is the actinic hazard, described in the international standard IEC 62471:2006 [5], which provides for a maximum daily dose of 230 J/m^2^ at 222 nm; 30 J/m^2^ at 270 nm; 60 J/m^2^ at 254 nm; and 272 kJ/m^2^ at 365 nm. The hazard function pertaining to wavelengths in the visible light spectrum (400 nm to 700 nm) is the blue light retinal hazard.

The UV-C actinic risks include erythema (reddening) of the skin, photokeratitis (irritation) of the eye, and low-level risk of long-term skin cancer. The health consequences of those three primary risks in the UV-C are minor, as summarized in CIE 187:2010 [47], excerpted here:

“Known side effects of overexposure to UV-C radiation include transient corneal and conjunctival irritation (photo-keratoconjunctivitis) and skin irritation (erythema), which disappear within a 24–48-hour period, not currently known to produce lasting biological damage.” “Using the best available information, a lifetime exposure risk was calculated which showed that an accumulated daily exposure to 254 nm radiation at the ACGIH^®^/ICNIRP threshold limit value (TLV^®^) (i.e., 6 mJ/cm^2^ [3 mJ/cm^2^ effective actinic]), received over 8 hours for 5 days a week and over 20 years, would increase the risk of non-melanoma skin cancer by a factor of about 0.37%.”

The UV-A risks to human skin and eyes have been thoroughly studied for decades. Exposure to UV-A alone can produce erythema, but only at very high radiant exposures (*i.e.*, 100 kJ/m^2^), as shown by more recent studies. The ACGIH [44] (and IEC 62471 [5]) guideline values are approximately 2 to 4 times less than these minimum erythemal dose (MED) values. The ELs were developed by considering lightly pigmented populations with greatest sensitivity and predisposition to adverse health effects from exposure to UV. ELs are not intended to apply to exposure of pathologically photosensitive individuals, to people concomitantly exposed to photosensitizing agents, or to neonates.

In any technical risk-benefit trade-off analysis, both the risks and the benefits should be reliably quantified to support the decision to employ the technology. DIBEL protocols offer well-established germicidal efficacy against target pathogens, promising significant benefit to public health, while directly exposing individuals to UV radiation at levels below well-established safety limits. Even in the unlikely event of accidental overexposure, the risks are also well established and demonstrated to be minor relative to the benefits of disease prevention.

### 7.3 Other Hazards of UV Light

The daily actinic exposure from UV-C DIBEL irradiation on human skin and eyes at the EL is generally equivalent to about 5 min or less of direct sunlight. Consequently, the effects of UV-C DIBEL on plants is also expected to be negligible, as for humans. Gradual fading or degradation of some especially susceptible materials is possible. In particular, exposure of sensitive art objects should be avoided. However, most typical interior materials used in fabrics, carpeting, and structural polymers are expected to exhibit little to no observable discoloration or mechanical degradation after exposures equivalent to many years of 254 nm irradiation below the ELs [48]. Degradation of materials inside of HVAC systems subject to high-intensity UV-C, far above the EL, has also been reported [49].

#### 7.3.1 UV-C Hazards to Plants

There is a body of literature on the use of UV-C for the inactivation of pests and microbes on plants, mostly at 254 nm, and more recently at shorter wavelengths from excimer lamps. The studies are generally performed with UV-C doses ranging from 500 J/m^2^ to 80 kJ/m^2^, whereas the daily EL for human actinic radiation at 254 nm is 30 J/m^2^. These studies therefore represent DIBEL exposures equivalent to exposure for about a month to several years. The scientific literature is relatively sparse, not including a UV-C action spectrum (damage *vs*. wavelength) nor covering irradiance levels as low as those of DIBEL. Examples of results reported include the following studies:

• Slight growth inhibition of ~20% was observed following a 1000 J/m^2^ dose of either 222 nm or 254 nm UV-C with *Arabidopsis* plants (a popular model organism in plant biology and genetics) [50].
• No phototoxic effects were observed at 1.2 W/m^2^ (~600× the EL of 2 mW/m^2^ at 254 nm) using a 248 nm excimer laser for the treatment of a spider pest and eggs on strawberry leaves [51].

To the contrary, several studies have demonstrated positive benefits of low doses of UV-C (and UV-B), such as:

• Strawberry plants that were inoculated with the causal agent of leaf spot disease and then repeatedly irradiated with UV-C for 5 weeks with a cumulative dose of 10.2 kJ/m^2^ (5 weeks of a DIBEL dose at 254 nm would be 1.05 kJ/m^2^) displayed reduced symptoms and increases in the activity of several defense enzymes [52].
• The treatment of UV-C on the leaves of three different strawberry cultivars applied at a rate of 3.4 kJ/m^2^ and 6.8 kJ/m^2^ (~ 100× DIBEL dose) every second day may improve resistance against mold without any apparent adverse impact on the plants [53].

Therefore, only qualitative expectations may be determined at this date, but it appears that a DIBEL dose is not likely to cause damage to plants.

## 8 Conclusions

Although it has been long used for disinfection purposes, UV radiation has mostly been limited to applications where humans are absent or shielded from the disinfecting source. DIBEL is a method of applying germicidal UV in such a way that occupied spaces may be directly disinfected. This is achieved by limiting UV to doses that are below industry-accepted exposure limits for repeated exposure of humans while simultaneously maintaining doses above those required for useful reductions of pathogenic organisms in the space.

DIBEL may be implemented with any emitter that provides wavelengths that are germicidal, from far UV-C to visible. It is, however, particularly attractive given the relatively recent availability of UV-C LEDs, which present a compact form factor coupled with sufficient radiated power. These products can be practically deployed unobtrusively in occupied spaces.

When applied to air disinfection, a resulting equivalent ACH_eq_ can be calculated for a given pathogen, allowing DIBEL to be quantitatively compared to, or added to, traditional methods of removing or inactivating aerosolized pathogens.

With consideration to the COVID-19 pandemic caused by the SARS-CoV-2 virus, an ACH_eq_ of 4 h^−1^ is presently achievable over a continuous 8 h period for SARS-CoV-2 with UV-C LEDs, and future improvements in LED technology and optics are anticipated to enable improvements up to 150 h^−1^ in the coming decade. DIBEL thus has the potential to be a useful tool in the battle against pathogens in occupied spaces, both now and in the future.

A future publication will provide experimental results of the efficacy of UV-C DIBEL against several target pathogens, validating the theoretical framework herein.
